# Contemporary update of cancer control after radical prostatectomy in the UK

**DOI:** 10.1038/sj.bjc.6602206

**Published:** 2004-11-02

**Authors:** M H Winkler, F A Khan, M Shabir, A Okeke, M Sugiono, P McInerney, G B Boustead, R Persad, A V Kaisary, D A Gillatt

**Affiliations:** 1 Royal Free Hospital, London, UK; 2 Southmead Hospital, Bristol, UK; 3 Derriford Hospital, Plymouth, UK; 4 Lister Hospital, Stevenage, UK; 5 Bristol Royal Infirmary, UK

**Keywords:** prostate neoplasm, adenocarcinoma, radical prostatectomy, prostate-specific antigen

## Abstract

Despite a significant increase of the number of radical prostatectomies (RPs) to treat organ-confined prostate cancer, there is very limited documentation of its oncological outcome in the UK. Pathological stage distribution and changes of outcome have not been audited on a consistent basis. We present the results of a multicentre review of postoperative predictive variables and prostatic-specific antigen (PSA) recurrence after RP for clinically organ-confined disease. In all, 854 patient's notes were audited for staging parameters and follow-up data obtained. Patients with neoadjuvant and adjuvant treatment as well as patients with incomplete data and follow-up were excluded. Median follow-up was 52 months for the remaining 705 patients. The median PSA was 10 ng ml^−1^. A large migration towards lower PSA and stage was seen. This translated into improved PSA survival rates. Overall Kaplan–Meier PSA recurrence-free survival probability at 1, 3, 5 and 8 years was 0.83, 0.69, 0.60 and 0.48, respectively. The 5-year PSA recurrence-free survival probability for PSA ranges <4, 4.1–10, 10.1–20 and >20 ng ml^−1^ was 0.82, 0.73, 0.59 and 0.20, respectively (log rank, *P*<0.0001). PSA recurrence-free survival probabilities for pathological Gleason grade 2–4, 5 and 6, 7 and 8–10 at 5 years were 0.84, 0.66, 0.55 and 0.21, respectively (log rank, *P*<0.0001). Similarly, 5-year PSA recurrence-free survival probabilities for pathological stages T2a, T2b, T3a, T3b and T4 were 0.82, 0.78, 0.48, 0.23 and 0.12, respectively (log rank, *P*=0.0012). Oncological outcome after RP has improved over time in the UK. PSA recurrence-free survival estimates are less optimistic compared to quoted survival figures in the literature. Survival figures based on pathological stage and Gleason grade may serve to counsel patients postoperatively and to stratify patients better for adjuvant treatment.

Recurrence of prostate cancer is of concern for men undergoing radical prostatectomy (RP) with curative intent. Nearly 30% of patients will develop prostatic specific antigen (PSA) recurrence. Patients having undergone RP and treating surgeons need to know the risk of PSA recurrence following RP. However, figures for PSA recurrence following RP in the UK are sparse (Feneley *et al*, 1996; Doherty *et al*, 2000; Bott and Kirby, 2002). This and a trend towards lower preoperative PSA values and stage over the last 10 years suggests there is a continuing but hitherto unmet need for reliable outcome data from large treatment series to document cancer control rates following RP with curative intend in the UK.

Many variables have been shown to be associated with the probability of PSA recurrence following RP. It is not entirely certain as to which variable indicates tumour recurrence for the individual patient. These variables may also change over time and may be of varying importance for different patient populations. Contemporary updates of variables predicting outcome will provide more accurate information for prognostic models and may aid clinical decision-making. In particular, the effect of a downward stage migration for prostate cancer may have an impact on PSA recurrence following RP. This stage migration is usually seen in regions with a high penetrance of PSA testing. It is estimated that only 3.5% of all men are tested annually with a serum PSA in the UK compared to 20% in the US in 1999 (Melia and Moss, 2001; Oliver *et al*, 2003).

Despite the absence of reliable evidence of its effectiveness, RP has become an increasingly popular treatment for selected patients with organ-confined prostate cancer in England over the last decade (Oliver *et al*, 2003). Holmberg *et al* (2002) showed in the only adequately powered randomised trial to date that prostate cancer-specific death is reduced by 50%, but there was no significant difference between surgery and watchful waiting in terms of overall survival (Holmberg *et al*, 2002). There is as yet no confirmed influence on the survival of men with screen-detected disease.

We retrospectively reviewed a large series of men who underwent RP in the South of England to document contemporary PSA recurrence-free survival probabilities and identify pathological factors of PSA recurrence.

## MATERIALS AND METHODS

In all, 854 patient's notes from five Urology units (two academic units and three District General Hospitals) were audited for preoperative staging parameters and follow-up data obtained. All units had at least a yearly throughput of 20 procedures. Data were collected retrospectively from 1989 to 1999. From 2000 onwards databases were continuously updated. Most patients underwent radical retropubic prostatectomy apart from 150 patients who underwent radical perineal prostatectomy. Patients with neoadjuvant treatment were included in the analysis of stage migration. Patients with neoadjuvant (84 patients) and adjuvant treatment prior to PSA recurrence (36 patients) as well as patients with incomplete data and follow-up of <12 months (29 patients) were excluded from survival analysis. Cox's proportional-hazards regression was used to identify factors associated with biochemical recurrence in the first 5 years following RP. Patients with positive lymph nodes were included if they underwent synchronous RP in the analysis of overall survival. However, since 150 patients underwent radical perineal prostatectomy and only 10 of those patients had synchronous lymphadenectomy, lymph node-positive patients were excluded from the multivariate analysis. Probability estimates of recurrence-free survival were calculated with the Kaplan–Meier method for all significant predictors. Continuity corrected *χ*^2^ test and Wilcoxon's test were used to compare factors reflecting patient selection. Log rank and Wilcoxon's test were used to compare Kaplan–Meier survival curves and survival probabilities. The preoperative PSA was recorded prior to any intervention, that is, digital rectal examination, biopsy or TURP. Biochemical recurrence was defined as at least two consecutive elevations above a PSA of 0.2 ng ml^−1^. A positive surgical margin was defined as prostatic adenocarcinoma at the inked margin of the pathological specimen regardless of the location of the margin and the extent of margin and carcinoma involved. This was necessary because histopathological reporting practice varied widely. Staging followed the 1997 TNM classification.

## RESULTS

Median prebiopsy PSA was 10.0 ng ml^−1^ (mean 12.3 ng ml^−1^) (Table 1

**Table 1:** Pre- and post-operative descriptive statistics

	**Frequency (*n*)**	**Relative %**
*PSA (ng ml*^−*1*^)^a^		
<4	63	8.6
4–10	320	43.8
10.1–20	249	34.1
>20	99	13.5
		
*Biopsy Gleason grade*^a^		
2–4	135	18.9
5, 6	436	61.3
7	102	14.3
8–10	38	5.3
		
*Clinical stage*^a^		
T1a/b	38	5.6
T1c	287	41.9
T2a	257	37.6
T2b	102	14.9
		
	**Frequency (*n*)**	**ng** **ml^−1^**
*PSA (all patients)*^a^		
Median	731	10.0
Mean	731	12.3
		
*PSA (pts without recurrence)*		
Median	551	9
Mean	551	10
		
*PSA (pts with recurrence)*		
Median	180	14
Mean	180	16.6
		
	**Frequency (*n*)**	**Relative %**
*Pathological stage*^a^		
T0	6	0.8
T2a	347	47.1
T2b	55	7.5
T3a	267	36.3
T3b	40	5.4
T4	21	2.9
		
*Pathological Gleason grade*^a^		
2–4	65	8.9
5, 6	402	55.1
7	189	25.9
8–10	74	10.1
Lymph node status^a^		
Negative	667	95.1
Positive	34	4.9
		
*Surgical margin status*^a^		
Negative	465	63.2
Positive	271	36.8

PSA=prostatic-specific antigen.

a Patients with missing data excluded.

). Pre- and postoperative descriptive data of the entire cohort of 747 patients after exclusion of 107 patients with missing data of interest for this analysis are shown in Table 1. A median age of 64 years was recorded. There was a statistically significant trend over time towards lower preoperative PSA (*χ*^2^=78.62, *P*<0.0001), improved pathological stage (*χ*^2^=12.64, *P*=0.0132) and improved local excision rates (*χ*^2^=10.65, *P*=0.0308). Surgically organ-confined disease was found in 39% in 1988–1994 and 61% in 2001–2002. A positive surgical margin was observed in 46% in 1988–1994 and 30% in 2001–2002.

The median follow-up was 52 months for all the 705 patients included in the survival analysis.

Overall Kaplan–Meier PSA recurrence-free survival probabilities at 1, 3, 5 and 8 years were 0.83 (CI 0.80–0.86), 0.69 (CI 0.64–0.74), 0.60 (CI 0.54–0.66) and 0.48 (CI 0.37–0.59), respectively. The median overall survival time was reached at 9.07 (CI 5.99–12.14) years.

**Figure 1:**
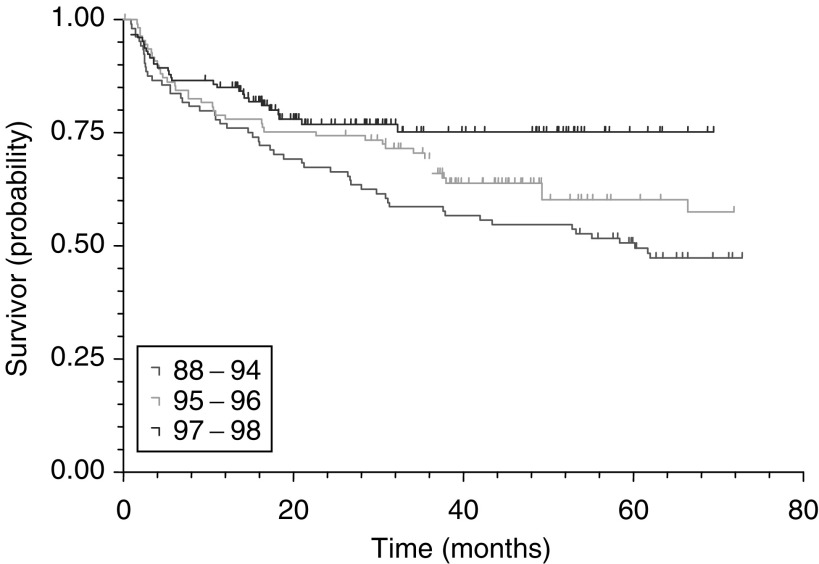
PSA recurrence-free survival curves by year of operation (log rank, *P*=0.0006).

The 5-year PSA recurrence-free survival probabilities increased significantly over time and were 0.49 (CI 0.40–0.58), 0.63 (CI 0.53–0.73) and 0.79 (CI 0.71–0.87) for the years 1988–1994, 1995–1996 and 1997–1998, respectively (log rank, *P*<0.0001) (Figure 1

). Survival probabilities could not be calculated for 1999–2002 because follow-up was too short to permit comparison over time.

**Figure 2:**
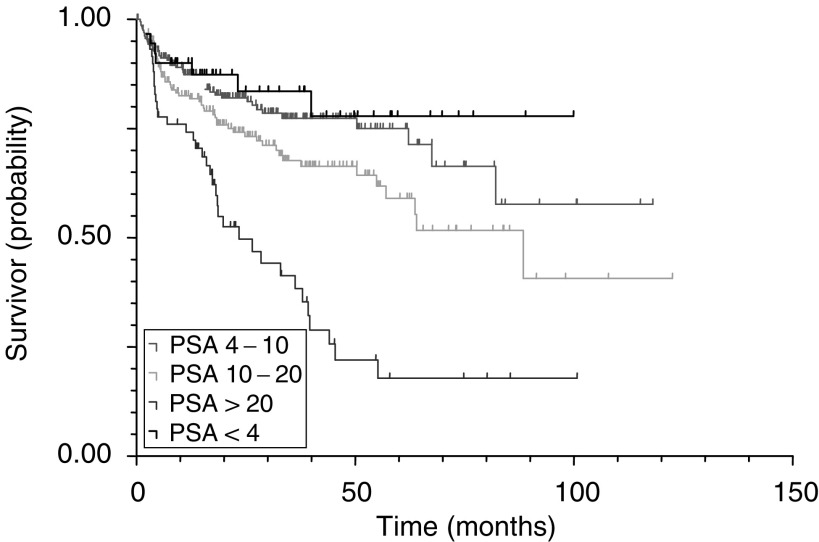
PSA recurrence-free survival curves by preoperative PSA (log rank, *P*<0.0001).

Cox's (proportional-hazards) regression identified preoperative PSA (*P*<0.0001), pathological Gleason grade (*P*=0.0178), pathological stage (<0.0001) and margin status (<0.0001) as significant predictors of PSA recurrence for the 671 patients without lymph node metastases and after other exclusions. Kaplan–Meier survival curves for PSA, pathological stage, pathological Gleason grade and surgical margin status are illustrated in Figures 2

, 3

**Figure 3:**
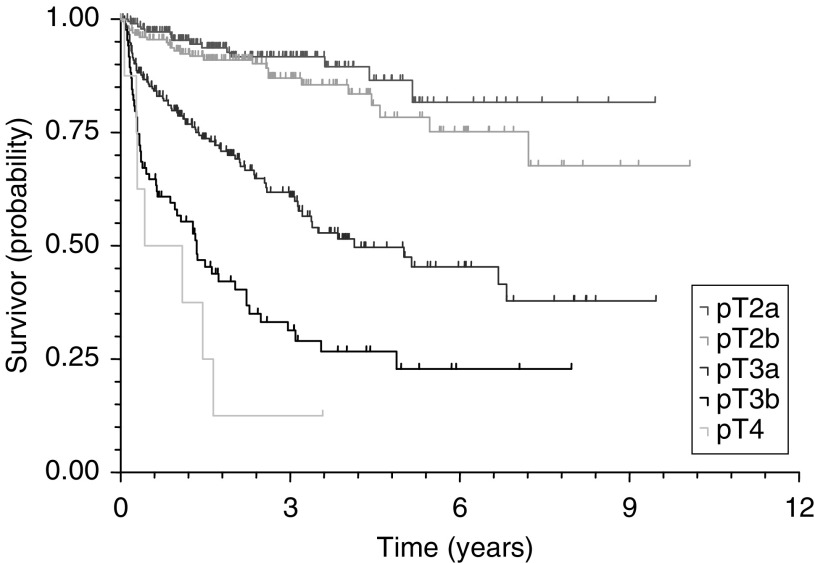
PSA recurrence-free survival curves by pathological stage (log rank, *P*<0.0001).

, 4

**Figure 4:**
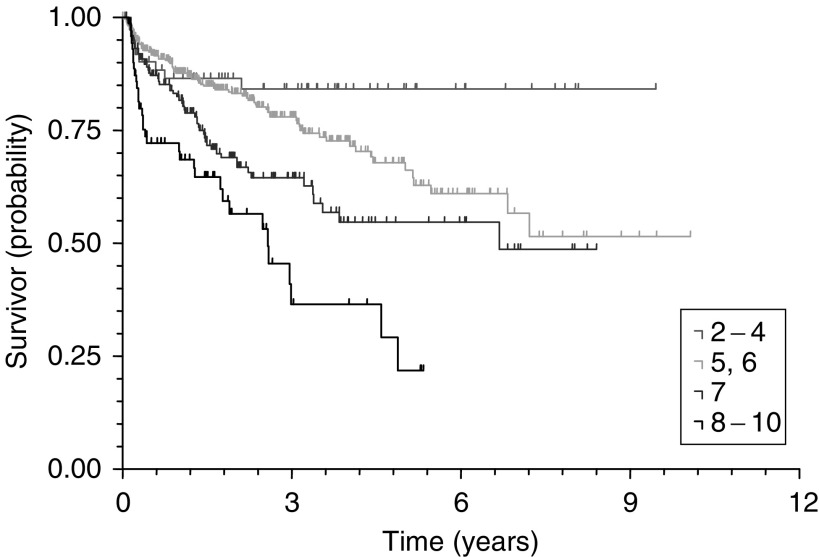
PSA recurrence-free survival curves by pathological Gleason grade (log rank, *P*<0.0001).

and 5

**Figure 5:**
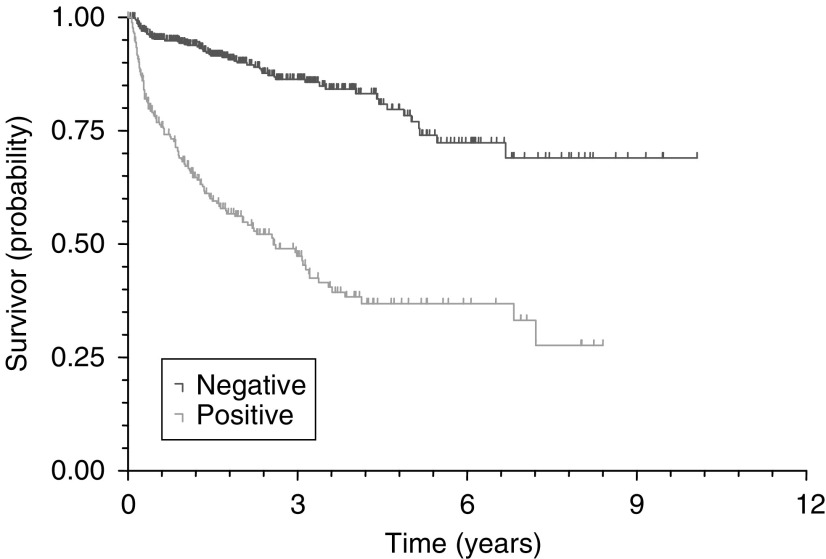
PSA recurrence-free survival curves by status of resection margin (log rank, *P*<0.0001).

. The 5-year PSA recurrence-free survival probabilities and median survival times for all significant predictive variables (PSA, pathological stage, pathological Gleason grade and margin status) are shown in Table 2

**Table 2:** The 5-year PSA recurrence-free survival probabilities and median PSA recurrence-free survival times for significant predictive variables

		**PSA recurrence-free probability**		**Median PSA survival time**	
**Variable**	***n***	**At 5 years**	**CI**	**Years**	**CI**
*Preop PSA*^a^					
<4	45	0.82	0.68–0.97	—	—
4–10	236	0.73	0.64–0.82	—	—
10–20	180	0.59	0.50–0.68	7.21	6.64–7.78
>20	58	0.20	0.07–0.33	2.57	1.69–3.46
					
*Pathological stage*					
T2a	191	0.82	0.70–0.94	—	—
T2b	205	0.78	0.70–0.82	—	—
T3a	216	0.48	0.38–0.58	4.13	1.88–6.37
T3b	85	0.23	0.11–0.35	1.35	0.91–1.79
T4	8	0.12	0.00–0.34	0.43	0.28–1.45
					
*Pathological Gleason grade*^a^					
2–4	52	0.84	0.74–0.94	—	—
5 and 6	387	0.66	0.58–0.74	—	—
7	180	0.55	0.44–0.66	6.67	3.38–8.40
8–10	72	0.21	0.03–0.39	2.57	1.84–3.30
					
*Margin status*					
Negative	449	0.78	0.72–0.84	—	—
Positive	256	0.37	0.29–0.46	2.57	1.85–3.29

PSA=prostatic-specific antigen; CI=confidence interval.

a Patients with missing data excluded.

.

## DISCUSSION

Despite a significant increase of the number of RPs to treat organ-confined prostate cancer, there is very limited documentation of its oncological outcome in the UK. Pathological stage distribution and changes of outcome have not been audited on a consistent basis. We present the results of a multicentre review of postoperative predictive variables and PSA recurrence after RP for clinically organ-confined disease.

Particular attention was paid to include all patients who had a RP regardless of surgical margin or lymph node status. However, the majority of patients who underwent radical perineal prostatectomy did so without pelvic lymphadenectomy. All patients with positive lymph nodes were therefore excluded from the multivariate analysis, but were included in the calculation of overall survival probabilities. True outcome figures based on preoperative parameters can only be calculated if an intention to treat analysis is used.

Traditionally, urologists in England have been slow to take up this procedure to cure prostate cancer because there was a distinct lack of reliable data to support any specific recommendation for the treatment of early prostate cancer. Recently, Oliver *et al* (2003) demonstrated a nearly 20-fold increase of the number of RPs performed annually between 1991 and 1999 in England. They stated that these developments occurred in the absence of robust information about the effectiveness of RP. There is now some limited evidence that RP can reduce death from prostate cancer (Holmberg *et al*, 2002).

We have shown that a significant stage migration has occurred in England with the result of large improvements in 5-year PSA recurrence-free survival after RP (Figure 1). Stage migration towards lower clinical and pathological stage has been demonstrated in other countries (Litwiller *et al*, 1995; Moul *et al*, 2002) and likewise this has led to an improvement in overall PSA recurrence-free survival rates (Moul *et al*, 2002; Ung *et al*, 2002; Freedland *et al*, 2003). The changes of pathological stage proportions have an impact on overall PSA recurrence-free survival. However, PSA recurrence-free survival rates for the separate stages and grades should only be affected marginally. Hence, the calculated 5-year PSA recurrence-free survival probabilities for stages, PSA ranges, Gleason grade groups and margins status should be reliable and useful reference data despite the observed stage migration.

It is encouraging to see urologists in England have largely moved away from selecting patients with a preoperative PSA of >20 ng ml^−1^ for RP with their associated high rates of nonorgan-confined disease (D’Amico *et al*, 2002). Rates of patients with a preoperative PSA >20 ng ml^−1^ have dropped from 38% in 1988–1994 to 7% in 2001–2002. PSA recurrence rates after RP for patients with a preoperative PSA of >20 ng ml^−1^ can be as high as 46–59% (Hull *et al*, 2002), being 80% in this study. The median time to PSA recurrence for this patient group is only 2.57 years (Table 2). Even patients with a preoperative PSA of 10–20 ng ml^−1^ only have a median time to PSA recurrence of 7.21 years and the PSA recurrence-free survival at 5 years is 59%. This is in contrast to published figures of 65–72% for PSA recurrence-free survival rates at 5 years (Hull *et al*, 2002). One has to question the use of a procedure with curative intent under these circumstances and preoperative selection criteria may have to be tightened up even further. If a patient with a PSA >20 ng ml^−1^ is selected for RP, he should certainly be counselled appropriately for an 80% probability of recurrence and the anticipated need for further treatment.

Although positive surgical margin rates have improved, a rate of 30% is still higher than the 14–19% reported in most other RP series (Stamey *et al*, 1998; Hull *et al*, 2002; Han *et al*, 2003). Although most authors have reported a reduction of the positive surgical margin rate as a reflection of improved case selection over time (Stamey *et al*, 1998; Han *et al*, 2003), others could not demonstrate this (Freedland *et al*, 2003). It may be that English urologists still operate on larger volume cancers because screening healthy asymptomatic men (and finding asymptomatic, low volume cancers) is currently discouraged by the Department of Health. Certainly, all surgeons involved in this audit have a high annual throughput of at least 20 procedures, which makes it less likely that this figure is due to technical shortfalls during the learning curve. However, surgeon volume does have an impact on outcome and is inversely related to in-hospital complications and length of stay in men undergoing RP, illustrating the potential influence of a learning curve (Hu *et al*, 2003). Finally, we used a wide definition of positive surgical margins in order to accommodate the variable reporting style of the involved histopathologists who were not always specialised uropathologists. This may have slightly inflated our positive surgical margin rates. It is of paramount importance to reduce the positive surgical margin rate with its associated high PSA recurrence rate (Table 2). This may mean that there should only be an attempt to spare the neurovascular bundles for highly selected cases.

Multivariate analysis identified preoperative PSA (*P*<0.0001), pathological Gleason grade (*P*=0.0178), pathological stage (<0.0001) and margin status (<0.0001) as highly significant predictors of PSA recurrence as already known (Hull *et al*, 2002). PSA recurrence-free survival figures are presented for these predictors. The PSA recurrence rates are in general higher for higher pathological stages and grades in comparison to the literature (Han *et al*, 2001, 2003; Hull *et al*, 2002). It is surprising to see pathological Gleason grade as the least significant predictor of PSA recurrence. There is only a minimal difference of PSA recurrence-free survival rates between Gleason grade 5–6 and 7 at 8 years. This suggests that many Gleason grade 7 cancers have probably been undergraded as a result of the varying reporting practice.

Overall PSA recurrence-free survival estimates are less optimistic compared to frequently quoted figures (Stamey *et al*, 1998; Walsh, 2000; Han *et al*, 2001, 2003; Hull *et al*, 2002) and seem to show a more realistic picture following surgery for organ-confined prostate cancer in the UK. Generally, a figure of 78–84% is quoted for overall PSA recurrence-free survival at 5 years compared to 60% in our study (Hull *et al*, 2002; Han *et al*, 2003). The 5-year PSA recurrence-free survival rates improved over time from 49% before 1994 to 79% in 1997–1998 (Figure 1). This is indeed an acceptable figure and corresponds well with other published PSA recurrence figures (Stamey *et al*, 1998), albeit the best series do report 5-year PSA recurrence-free survival rates of >80% (Han *et al*, 2003). It appears that the combination of improved case selection and increasing use of PSA testing lead to an improvement of PSA recurrence-free survival after RP for organ-confined prostate cancer in the UK.
